# Effects of *Plasmodium falciparum* infection on umbilical artery resistance and intrafetal blood flow distribution: a Doppler ultrasound study from Papua New Guinea

**DOI:** 10.1186/s12936-017-1689-z

**Published:** 2017-01-19

**Authors:** Maria Ome-Kaius, Stephan Karl, Regina Alice Wangnapi, John Walpe Bolnga, Glen Mola, Jane Walker, Ivo Mueller, Holger Werner Unger, Stephen John Rogerson

**Affiliations:** 1 Papua New Guinea Institute of Medical Research (PNG IMR), Madang, Papua New Guinea; 2 Population Health and Immunity Division, Walter and Eliza Hall Institute of Medical Research (WEHI), 1G Royal Parade, Parkville, 3052 Australia; 3 Department of Medical Biology, University of Melbourne, Parkville, VIC Australia; 4 Department of Obstetrics and Gynaecology, Modilon General Hospital, Madang, Papua New Guinea; 5 Department of Obstetrics and Gynaecology, University of Papua New Guinea, Port Moresby, Papua New Guinea; 6 Department of Radiology, Royal Infirmary of Edinburgh, 51 Little France Crescent, Edinburgh, EH16 4SA UK; 7 Institut Pasteur, 28 Rue de Dr. Roux, 75015 Paris, France; 8 Department of Obstetrics and Gynaecology, Royal Infirmary of Edinburgh, 51 Little France Crescent, Edinburgh, EH16 4SA UK; 9 Department of Medicine (Royal Melbourne Hospital), The University of Melbourne, Post Office Royal Melbourne Hospital, Parkville, VIC 3050 Australia

**Keywords:** Umbilical artery resistance, Middle cerebral artery pulsatility, Cerebroplacental ratio, Doppler, Sub-microscopic, Fetal growth

## Abstract

**Background:**

Doppler velocimetry studies of umbilical artery (UA) and middle cerebral artery (MCA) flow help to determine the presence and severity of fetal growth restriction. Increased UA resistance and reduced MCA pulsatility may indicate increased placental resistance and intrafetal blood flow redistribution. Malaria causes low birth weight and fetal growth restriction, but few studies have assessed its effects on uteroplacental and fetoplacental blood flow.

**Methods:**

Colour-pulsed Doppler ultrasound was used to assess UA and MCA flow in 396 Papua New Guinean singleton fetuses. Abnormal flow was defined as an UA resistance index above the 90th centile, and/or a MCA pulsatility index and cerebroplacental ratio (ratio of MCA and UA pulsatility index) below the 10th centile of population-specific models fitted to the data. Associations between malaria (peripheral infection prior to and at ultrasound examination, and any gestational infection, i.e., ‘exposure’) and abnormal flow, and between abnormal flow and birth outcomes, were estimated.

**Results:**

Of 78 malaria infection episodes detected before or at the ultrasound visit, 62 (79.5%) were *Plasmodium falciparum* (34 sub-microscopic infections), and 16 were *Plasmodium vivax*. *Plasmodium falciparum* infection before or at Doppler measurement was associated with increased UA resistance (adjusted odds ratio (aOR) 2.3 95% CI 1.0–5.2, *P* = 0.047). When assessed by ‘exposure’, *P. falciparum* infection was significantly associated with increased UA resistance (all infections: 2.4, 1.1–4.9, *P* = 0.024; sub-microscopic infections 2.6, 1.0–6.6, *P* = 0.051) and a reduced MCA pulsatility index (all infections: 2.6, 1.2–5.3, *P* = 0.012; sub-microscopic infections: 2.8, 1.1–7.5, *P* = 0.035). Sub-microscopic *P. falciparum* infections were additionally associated with a reduced cerebroplacental ratio (3.64, 1.22–10.88, *P* = 0.021). There were too few *P. vivax* infections to draw robust conclusions. An increased UA resistance index was associated with histological evidence of placental malaria (5.1, 2.3–10.9, *P* < 0.001; sensitivity 0.26, specificity 0.93). A low cerebroplacental Doppler ratio was associated with concurrently measuring small-for-gestational-age, and with low birth weight.

**Discussion/conclusion:**

Both microscopic and sub-microscopic *P. falciparum* infections impair fetoplacental and intrafetal flow, at least temporarily. Increased UA resistance has high specificity but low sensitivity for the detection of placental infection. These findings suggest that interventions to protect the fetus should clear and prevent both microscopic and sub-microscopic malarial infections.

*Trial Registration* ClinicalTrials.gov NCT01136850. Registered 06 April 2010

**Electronic supplementary material:**

The online version of this article (doi:10.1186/s12936-017-1689-z) contains supplementary material, which is available to authorized users.

## Background

The identification and management of risk factors for fetal growth restriction (FGR) is key to optimizing pregnancy outcomes. The incidence of low birth weight [(LBW) <2500 g], FGR and infant death are substantially higher in low- and middle-income countries (LMICs) [1, 2].

*Plasmodium falciparum* and *Plasmodium vivax* infections are common during pregnancy in many LMICs, and are associated with LBW [3]; infection at any stage of pregnancy may affect birth weight and fetal growth [4–6]. Mechanisms by which malarial infection may cause FGR include deleterious effects on trophoblast migration and invasion capacity [5], placental vascular insufficiency through suppression of pro-angiogenic factors and reduced placental vessel development [7], impaired nutrient transport, and through causing imbalances in growth hormone levels [6, 8, 9].

Doppler ultrasound allows for non-invasive real-time assessment of uteroplacental and fetoplacental blood flow characteristics and intrafetal blood flow distribution. An increased umbilical artery resistance index (UARI) or absent/reversed UA end-diastolic flow in the second half of pregnancy indicates increased placental vascular resistance and uteroplacental insufficiency, due to poor villous angiogenesis or acute pathologies such as placental abruption [10]. A decrease in middle cerebral artery pulsatility index (MCAPI) in third trimester indicates blood flow redistribution to the fetal brain as a physiological adaptation to placental insufficiency [11]. This is an adaptive response to maintain brain growth at the expense of other body parts when oxygen and nutrient supply is limited, resulting in asymmetrical growth restriction [12]. UA and MCA interrogations predict adverse outcomes best amongst pregnancies measuring small-for-gestational-age [(SGA), <10th centile of a growth standard] or with decreasing growth centiles, both fetal size indicators of FGR [13].

A small number of ultrasound-based studies in LMICs have examined factors associated with abnormal placental development and fetal growth, and have evaluated the potential effects of malaria parasitaemia on uteroplacental and fetoplacental blood flow [14–19]. Symptomatic falciparum malaria in early third trimester was associated with acutely increased UARI and reduced MCA resistance in French Guiana [14, 15]. In Kenyan women in third trimester, concurrent malaria parasitaemia was associated with increased uterine artery resistance indices, but effects on UARI were not measured [16]. In another Kenyan cohort, increased UARI was seen amongst women with concurrent infection before 26 gestational weeks [17]. Infection in early pregnancy (<20 gestational weeks) was associated with increased uterine artery resistance in undernourished Congolese women, and with reduced UARI in later pregnancy amongst primigravidae, the latter perhaps being due to adaptive villous angiogenesis following a treated infection [18]. In a case–control study of *P. vivax* in pregnancy in Brazil, parasitaemia early in pregnancy was associated with reduced fetal growth later in pregnancy, but UARI did not differ between infected/uninfected groups [19].

In rural coastal Papua New Guinea (PNG), both *P. falciparum* and *P. vivax* infection are endemic [20], and malaria is an important risk factor for LBW [21], together with maternal betel (areca) nut consumption [22], macronutrient undernutrition [23], and tobacco smoking [24]. Here, macronutrient undernutrition and anaemia were observed to be key risk factors for measuring SGA and for poor fetal weight gain [25]. In the same study, malaria parasitaemia was associated with SGA in univariable but not in adjusted analyses. To date, the relationship between malaria infection and fetoplacental and intrafetal blood flow has not been evaluated in PNG.

This study evaluated the impact of *P. falciparum* and *P. vivax* infection as well as other potential preventable and treatable risk factors for LBW and FGR, such as undernutrition and anaemia, on fetoplacental (UA) and MCA Doppler flow indices and fetal size in a cohort of pregnant women co-enrolled in a large malaria prevention study in PNG.

## Methods

### Study setting and population

The study cohort consisted of women who participated in both a randomized controlled trial investigating the impact of intermittent preventive treatment (IPTp) with azithromycin (AZ) plus sulfadoxine–pyrimethamine (SP) on birth weight [23] and a nested ultrasound study evaluating factors associated with reduced fetal size and fetal weight gain in PNG [25]. The studies were conducted between November 2009 and February 2013 at nine health centres in Madang and Sumkar districts situated on the north coast of PNG.

All participants provided written informed consent for participation in the original trial and the ultrasound study. The research was approved by the PNG Institute of Medical Research, the PNG Medical Research Advisory Council and the Melbourne Health Human Research Ethics Committee, as previously described [23, 26].

The study area is characterized by perennial transmission of *P. falciparum* and *P. vivax*, and infant and adult macronutrient undernutrition is common; malarial infection, anaemia and undernutrition are important risk factors for FGR and LBW [21, 25, 27]. Up to 20% of newborns are LBW, which is an important contributor to PNG’s high infant mortality rate of 47.4 per 1000 live births in 2013 [28].

Women aged 16–49 years with no co-morbidities, a fundal height of 26 cm or less above the symphysis pubis, and who resided near to participating antenatal clinics, were invited to join the parent trial following screening at their first antenatal clinic visit [23]. Dating ultrasound scans were performed at or shortly after enrolment when possible. Trial participants with a singleton pregnancy and a dating ultrasound before 25 completed gestational weeks were eligible for inclusion in the present study [29].

At enrolment sociodemographic and clinical characteristics were recorded, anthropometric indicators of nutritional status were evaluated, and haemoglobin (Hb) was measured (HemoCue Ltd., Angelhom, Sweden). As per national guidelines, moderate-to-severe anaemia (Hb < 90 g/L) was treated with iron/folate supplements and albendazole, malaria with quinine (first trimester) or artemether–lumefantrine, and insecticide-treated bed nets were provided [30].

Women were subsequently invited to attend further ultrasound scan sessions during which fetal size parameters were measured and UA and MCA flow was interrogated. Women with no Doppler data, or who had a stillborn or congenitally abnormal baby, were not included in the study cohort [31].

### Clinical, sonographic and laboratory evaluations

At enrolment, information on educational and socio-economic status as well as risk behaviours such as tobacco smoking and betel (areca) nut chewing was obtained. Maternal anthropometrics such as height, weight and mid-upper arm circumference (MUAC) were recorded, and pulse, temperature and blood pressure were measured.

Maternal blood was collected for diagnostic purposes during subsequent scheduled and unscheduled visits, and birth weight (BW) was determined using a digital scale (Charder Medical, Taiwan, accuracy: 10 g).

Malaria infection was diagnosed by light microscopy (LM) and quantitative real-time polymerase chain reaction (qPCR), using established methodologies [32, 33]. Placental malaria on histology was classified as active (presence of parasitized cells) and past (haemozoin only) [34]. Presence of inflammation at enrolment was assessed by using a high-sensitive enzyme-linked immunosorbent assay for C-reactive protein (hs-CRP) (RD Systems, Minneapolis, USA).

Transabdominal ultrasound scans were performed using a portable ultrasound scanner (Logiqbook XP, General Electric Medical Systems, UK). The crown-rump length (<13 gestational weeks) or head circumference (HC) (femur length (FL) if unavailable) were used to estimate GA (gestational age), in accordance with published guidelines [30]. The study did not use other estimators of GA, such as last menstrual period, given their poor performance in this and similar cohorts [35, 36].

A colour-pulsed Doppler ultrasound, using a 2–5 MHz convex abdominal probe, was performed to assess UA and MCA flow. Measurements were performed in the absence of fetal breathing and body movement, and were recorded over a minimum of three uniform heart cycles. UA Doppler flow was assessed on a free loop of cord. Absent or reverse end-diastolic flow was not observed in the cohort. MCA flow was interrogated from near field MCAs only using a transtemporal view: flow velocity waveforms were obtained from the area where the MCA joins the Circle of Willis. Fetal abdominal circumference (AC), HC and FL were measured as previously described [26]. Fetal weights were estimated using a formula developed by Hadlock et al. [37] using a combination of all three measurements (or AC plus FL when HC could not be measured adequately due to fetal position, n = 9). A random sample of image stills were sent for external quality control. Of 90 randomly selected UA Doppler stills, all were deemed of good quality (adequate cord position, adequate and good quality wave obtained, adequate Doppler beam placement). Quality control criteria and results of fetal size parameters for this cohort have previously been reported [26].

### Exposures and outcome measures

Exposure variables to be examined a priori were selected based on the literature and findings from other secondary analyses of the parent trial. These included malaria infection, anthropometric indicators of women’s nutritional status, risk behaviour (tobacco smoking, betel nut chewing, alcohol consumption), socio-economic markers, reported educational attainment, and malaria prevention regimen received.

Protein-energy undernutrition was defined as low body mass index (BMI < 18.5 kg/m^2^) or low MUAC (<22 cm) at enrolment, and short stature as height <150 cm [38]. Moderate-to-severe anaemia was defined as Hb <90 g/L at enrolment, and inflammation as a hs-CRP level ≥5 mg/L. These exposures were evaluated at study enrolment only.

Malaria infection was defined as positivity by light microscopy and/or qPCR. Speciation into *P. falciparum* or *P. vivax* was based on qPCR results [33]. Malaria infections were categorized into microscopic and sub-microscopic *P. falciparum* infections and *P. vivax* infections. Due to the low number of *P. vivax* infections, not all statistical analyses could be performed for this species.

Analyses were conducted for infections preceding or coinciding with Doppler ultrasounds in order to assess the effect of malaria infection on the subsequent/contemporaneous measurement. Most infections were detected at study enrolment. In addition, the analysis considered all infections detected (including those at delivery or after a Doppler scan) as a measure of exposure to malaria in general (similar to a ‘force of infection’ estimate). This is reasonable, as it can be expected that a considerable proportion of asymptomatic and low-level infections are only intermittently detected by PCR. Previous analyses of longitudinal cohort infection data has shown that PCR detection of infection is imperfect [39]. In pregnancy, this may be more pronounced as a result of placental sequestration. In addition, malaria transmission intensity on the north coast of PNG can vary considerably between villages [40]: it is likely a proportion of women is frequently exposed to infection, while the majority of women are not exposed to infection at all. As such, the detection of malaria infection during pregnancy is likely to be associated with undetected infection instances in the same woman at other study contacts, including those at or before Doppler scan. Analyses were also conducted considering only infections that were detected in the first 20 weeks of pregnancy.

Outcome variables were UARI [calculated as (peak systolic velocity − end diastolic velocity)/peak systolic velocity], MCAPI (calculated as (peak systolic velocity − end diastolic velocity)/time averaged velocity [41]), and cerebroplacental Doppler ratio (CPR), which is the ratio of the MCAPI and the UA pulsatility index.

For each, z-scores were calculated, to adjust for variations in GA at the time of the study, because fetoplacental and cerebral blood flow vary with GA [10, 11]. Previous research indicates that ethnic differences in fetoplacental flow may be limited [42], but sex-specific differences in umbilical and cerebral blood flow may exist [43].

Lastly, associations of measures indicating aberrant fetal growth with the Doppler measurements were evaluated. Specifically associations of Doppler indices with suspected SGA episodes at the time of scan, LBW, and placental malaria were investigated.

### Statistical analysis

Statistical analyses were performed using Stata 12.0 (StataCorp, USA) and Mathematica 9.0 (Wolfram Inc., USA). The majority of MCAPI and CPR measurements fell outside the previously published reference ranges, (see Additional file 1). As such, an abnormal MCAPI and CPR was defined as below the 10th centile, and abnormal UARI as above the 90th centile, of ranges determined in this population. These ranges were derived by fitting linear mixed effects models using the fractional polynomial approach described by Royston et al. [44], which accounts for repeat measurements per woman. Response variables (i.e., UARI, MCAPI and CPR) were transformed if required as indicated by Box-Cox regression of the left-hand side of the model. Measurements outside of these 10th/90th centile bands were selected using z-scores (10th/90th centiles are equivalent to z-score of ±1.28). In addition data analysis was performed using a categorical cut <1.0 to define an abnormal CPR.

To account for repeat measurements, associations were estimated using generalized estimating equations (GEE) with an exchangeable working correlation structure (binary outcomes) [45]. Analyses were adjusted a priori for fetal sex [18], gravidity and GA. Interactions (effect measure modification) were tested for nutritional indicators (BMI, MUAC, short stature), anaemia, inflammation, gravidity, and fetal sex as well as potential risk behaviour (tobacco smoking, betel nut chewing and alcohol) using multiplicative terms (equivalent to factorial interactions). *P* < 0.15 for the interaction term was used to define presence of effect measure modification.

## Results

### Baseline characteristics

The study cohort consisted of 396 singleton pregnancies that resulted in a congenitally normal live birth. The mean GA at enrolment was 19.6 weeks (Table 1). More than half of the women were primigravidae (53.3%), most lived rurally and 89 (22.47%) had a Hb reading below 90 g/L at enrolment (Table 1).

**Table 1:** Characteristics of study participants (N = 396)

Characteristic	N/total (%) or mean [SD]
Age (years)	24.3 (5.3)
Rural residence	239/381 (62.7)
Literate	375/396 (93.7)
Smoker	82/396 (20.7)
Areca nut user	320/395 (81.0)
Number of previous pregnancies	
0	211/396 (53.3)
1	87/396 (22.0)
≥2	98/396 (24.8)
Gestational age at recruitment (weeks)	19.6 (3.8)
Peripheral parasitaemia at recruitment by microscopy	19/396 (4.6)
Low mid-upper arm circumference	53/396 (13.4)
Low body mass index	17/396 (4.3)
Moderate-to-severe anaemia	89/396 (22.5)
Delivery outcomes	
Female newborn sex	212/395 (53.7)
Low birthweight (<2500 g)	67/396 (16.9)
Placental malaria on histology^a^	46/308 (14.9)

^a^Active and past placental infection

The 396 women were tested a total of 1565 times for malaria with either PCR or LM, 72 women tested positive for malaria infection in peripheral blood once, 13 twice and two women tested positive three times. Eighteen *P. falciparum* and seven *P. vivax* infections were detected prior to 20 completed weeks of gestation. Three infections episodes (all *P. falciparum*) were clinical.

A total of 1179 peripheral malaria infection screening episodes preceded or coincided with Doppler scans. Overall, 59 were positive for *P. falciparum* by LM or qPCR, and 27 infections were sub-microscopic. A total of 16 *P. vivax* infections were detected by either LM or qPCR (one LM and qPCR, 15 by qPCR only).

### Doppler velocimetry measurements

Of 396 women, 361 had UA Doppler studies: 282 had one, 67 had two and 12 had three studies (total: 452). MCA studies were performed on 298 women who had 370 MCA Doppler scans: 235 women were studied once, 55 women twice, seven women three times, and one woman four times. CPR data was available for 252 women, who had 303 paired UA and MCA studies. Of these, 206 women were studied once, 41 women twice and five women three times.

### Regression of Doppler velocimetry indices versus gestational age

The best fit equations (mean and standard deviations) for the relationship between UARI (Eqs. 1a, 1b), MCAPI (Eqs. 2a, 2b) and CPR (Eqs. 3a, 3b) with GA (weeks) are given below, where µ (a) are means and σ (b) are standard deviations. The equations were used to determine potentially abnormal values based on a 10th/90th centile cut-off (z = 1.28).

1a $$\upmu_{\text{UARI}} \left[ {\text{X}} \right] = 0.12220 + 7.01831{\text{X}}^{ - 1} ;{\text{ X}} = {\text{GA}}^{2}$$

1b $$\upsigma_{\text{UARI}} \left[ {\text{X}} \right]^{2} = 0.01905 + 3.89066{\text{ X}}^{ - 2} - 0.41972{\text{ X}}^{ - 1} ;$$

2a $$\upmu_{\text{MCAPI}} \left[ {\text{X}} \right] = - 0.4569 + 0.298861{\text{ Sqrt}}\left[ {\text{X}} \right];{\text{ X}} = {\text{GA}}^{0.6835705}$$

2b $$\upsigma_{\text{MCAPI}} \left[ {\text{X}} \right]^{ 2} = 1. 3 5 6 1- 0. 4 8 8 4 2 3 {\text{ Sqrt}}\left[ {\text{X}} \right] + 0.0 4 5 5 2 1 4 {\text{ X}}$$

3a $$\upmu_{\text{CPR}} \left[ {\text{X}} \right] = 0.287651 + 0.0291729{\text{ X}};{\text{ X}} = {\text{GA}}^{0.4537937}$$

3b $$\upsigma_{\text{CPR}} \left[ {\text{X}} \right]^{2} = 0.0288508 - 0.00101161{\text{ X}} + 0.0000351{\text{ X}}^{2}$$

**Fig. 1:**
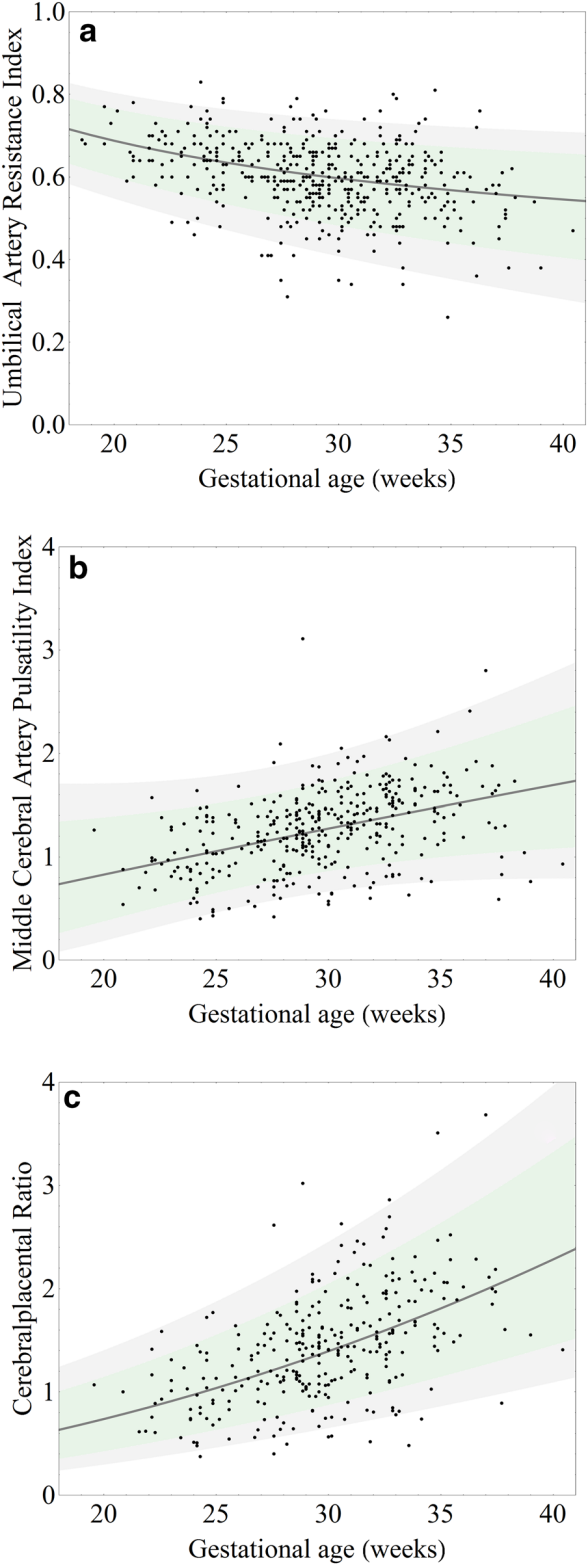
Umbilical artery resistance index (**a**), middle cerebral artery pulsatility index (**b**) and cerebroplacental Doppler ratio (**c**) change with gestational age and regression curves. *Black lines* are the means (µ) of the mixed effects model regression (given in Eqs. 1a, 2a, 3a). The *shaded areas* are the intervals between which 95% (*gre*y) and 80% (*light green*) of the data are located, equivalent to the 2.5th/97.7th and 10th/90th centiles and corresponding to Eqs. (1b, 2b, 3b) calculated as per Royston [18]

The Doppler velocimetry data and the respective regression curves and confidence intervals are shown in Fig. 1.

### Impact of malaria infection on Doppler velocimetry measurements

Tables 2, 3, 4 show the associations of malaria infections with the Doppler velocimetry measurements. Associations with other characteristics such as nutritional and socio-economic indicators, IPTp consumption, betel nut chewing, alcohol drinking and tobacco smoking, were not significant and are given in Additional files 2, 3 and 4.

**Table 2:** Association of peripheral blood malaria infection with elevated umbilical artery resistance index (>90th centile)

	% (N)	OR (95% CI)	P	aOR (95% CI)^a^	P
Peripheral malaria infection prior to Doppler scan^b^					
Any species	13.9 (63/452)	1.8 (0.8–3.9)	0.17	1.7 (0.8–3.9)	0.20
*P.f.*	11.5 (52/452)	2.3 (1.0–5.1)	0.048	2.3 (1.0–5.2)	0.047
*P.v.*	3.1 (14/452)	–	–	–	–
*P.f.* (sub)	4.8 (21/442)	2.3 (0.7–7.3)	0.15	2.1 (0.7–6.5)	0.20
Peripheral malaria infection at any stage in pregnancy (as a measure of exposure)^c^					
Any species	21.1 (76/361)	2.0 (1.0–4.0)	0.05	1.9 (0.9–4.0)	0.07
*P.f.*	16.6 (60/361)	2.4 (1.1–4.9)	0.021	2.4 (1.1–4.9)	0.024
*P.v.*	6.7 (24/361)	1.6 (0.6–4.9)	0.37	1.6 (0.5–5.0)	0.42
*P.f.* (sub)	7.4 (26/350)	2.7 (1.1–7.0)	0.04	2.6 (1.0–6.6)	0.05
Cumulative malaria detections with any species (as measure of exposure)^c^					
1	17.5 (63/361)	1.8 (0.9–4.0)	0.12	1.8 (0.8–3.9)	0.15
≥2	3.6 (13/361)	2.6 (0.7–9.2)	0.14	2.6 (0.7–9.8)	0.15

*P.f*., *P. falciparum*; *P.v*., *P. vivax*; sub, sub-microscopic

^a^Adjusted for fetal sex, gravidity and gestational age

^b^N (%) represents the number of scans

^c^N (%) represents the number of women

**Table 3:** Association of peripheral malaria infection with reduced fetal middle cerebral artery pulsatility index (<10th centile)

	% (N)	OR (95% CI)	P	aOR (95% CI)^a^	P
Peripheral malaria infection prior to Doppler scan^b^					
Any species	14.8 (55/370)	1.5 (0.6–3.5)	0.35	1.4 (0.6–3.2)	0.42
*P.f.*	12.7 (47/370)	1.9 (0.8–4.5)	0.15	1.8 (0.8–4.2)	0.17
*P.v.*	3.2 (12/370)	–	–	–	–
*P.f.* (sub)	6.6 (24/362)	1.5 (0.5–4.5)	0.52	1.4 (0.5–4.2)	0.59
Peripheral malaria infection at any stage in pregnancy (as a measure of exposure)^c^					
Any species	22.8 (68/298)	1.9 (0.9–3.9)	0.09	1.8 (0.9–3.79)	0.10
*P.f*.	18.1 (54/298)	2.6 (1.3–5.5)	0.010	2.6 (1.2–5.3)	0.012
*P.v*.	7.1 (21/298)	0.5 (0.1–4.0)	0.53	0.5 (0.1–3.9)	0.51
*P.f.* (sub)	9.4 (27/287)	2.9 (1.1–7.5)	0.027	2.8 (1.1–7.5)	0.035
Cumulative malaria detections with any species (as a measure of exposure)^c^					
1	18.8 (56/298)	1.2 (0.5–2.8)	0.63	1.2 (0.5–2.7)	0.69
≥2	4.0 (12/298)	6.2 (1.8–21.5)	0.004	6.0 (1.7–20.8)	0.005

*P.f*., *P. falciparum*; *P.v*., *P. vivax*; sub, sub-microscopic

^a^Adjusted for fetal gender, gravidity and gestational age

^b^N (%) represents the number of scans

^c^N (%) represents the number of women

**Table 4:** Association of peripheral malaria infection with reduced cerebroplacental Doppler ratio (<10th centile)

	% (N)	OR (95% CI)	P	aOR (95% CI)^a^	P
Any malaria infection prior to Doppler scan^b^					
Any species	14.8 (45/303)	1.7 (0.7–4.37)	0.25	1.7 (0.7–4.3)	0.25
*P.f.*	12.5 (38/303)	2.2 (0.9–5.7)	0.09	2.2 (0.8–5.5)	0.11
*P.v*.	3.3 (10/303)	–	–	–	–
*P.f*. (sub)	5.7 (17/296)	2.5 (0.7–8.2)	0.14	2.4 (0.7–8.2)	0.16
Peripheral malaria infection at any stage in pregnancy (as a measure of exposure)^c^					
Any species	21.4 (54/252)	1.6 (0.7–3.6)	0.29	1.6 (0.7–3.7)	0.29
*P.f*.	17.1 (43/252)	2.2 (0.9–5.0)	0.08	2.1 (0.9–5.0)	0.09
*P.v.*	7.1 (18/252)	1.1 (0.2–5.2)	0.91	1.2 (0.3–5.5)	0.84
*P.f*. (sub)	8.2 (20/244)	3.7 (1.3–10.7)	0.014	3.6 (1.2–10.9)	0.021
Cumulative malaria detections with any species (as a measure of exposure)^c^					
1	17.9 (45/252)	0.9 (0.3–2.5)	0.85	0.9 (0.3–2.5)	0.86
≥2	3.6 (9/252)	6.7 (1.6–27.5)	0.008	6.17 (1.5–25.5)	0.012

*P.f*., *P. falciparum*; *P.v*., *P. vivax*; sub, sub-microscopic

^a^Adjusted for fetal gender, gravidity and gestational age

^b^N (%) represents the number of scans

^c^N (%) represents the number of women

There was a significant association between the presence of *P. falciparum* parasitaemia before or at time of Doppler interrogation and a raised UARI measurement [adjusted odds ratio (aOR) 2.3, 95% CI 1.0–5.2; P = 0.047] (Table 2 and Additional file 5). When all peripheral infections were evaluated (before, during and after the Doppler measurements), similar, if not stronger effects were noted. *Plasmodium falciparum* infection during study participation was significantly correlated with having an elevated UARI measurement (>90th centile) [2.4 (95% CI 1.1–4.9); P = 0.024] (Table 2). In addition, there was a tendency towards an increased UARI amongst women who had only sub-microscopic *P. falciparum* infection (2.6, 1.00–6.6; P = 0.053). Malaria infections in the first 20 weeks of pregnancy were not associated with abnormal Doppler measurements.

There were no statistically significant associations between peripheral infections detected prior to or at the scan episode and reduced MCAPI. When all peripheral infections detected during pregnancy were considered, *P. falciparum* parasitaemia was associated with reduced MCAPI (2.7, 1.2–5.3; P = 0.012); a similar association was observed for sub-microscopic *P. falciparum* infections (Table 3), and the odds of abnormal MCAPI measurements increased with the number of infections detected (6.0, 1.7–20.8; P = 0.005).

There were no statistically significant associations of malaria infection with a reduced CPR when only peripheral infections detected prior to or at scan were analysed, although the association with previous *P. falciparum* infection approached significance (P = 0.11) (Table 4). However, there was a tendency towards an association between exposure to *P.* *falciparum* and a reduced CPR (P = 0.09, Table 4). In particular, sub-microscopic infections (3.6, 1.2–10.9, P = 0.021) and cumulative parasite detection (6.2, 1.5–25.5; P = 0.012) were associated with increased risk of reduced CPR. When a cut-off of 1.0 was used to define an abnormal CPR only cumulative parasite detection remained associated with an increased risk of an abnormal CPR (P = 0.024) (Additional file 6).

### Association of abnormal Doppler velocimetry with SGA, LBW and placental malaria

There were 50 episodes of SGA (estimated fetal weight <10th centile of the Hadlock reference standard) at the time of Doppler ultrasound in the cohort, 67 newborns (16.9%) had LBW and 46 out of the 308 histological analyses (14.9%) indicated placental infection (past and active infection).

Table 5 shows the associations between Doppler velocimetry measurements and measuring SGA at the time of scan, LBW (<2500 g) or placental infection.

**Table 5:** Doppler velocimetry measurements and associations with measuring small for gestational age at delivery, low birth weight and placental infection on histology

	% (N)	OR (95% CI)	*P*	aOR (95% CI)*	P*
Small for gestational age (n = 50)^b^					
Umbilical artery resistance index >90th centile	12.0 (44/368)	1.3 (0.4–4.0)	0.65	1.2 (0.4–4.1)*	0.73
Middle cerebral artery pulsatility index <10th centile	11.6 (34/293)	2.0 (0.7–5.4)	0.16	2.3 (0.8–6.1)	0.10
Cerebroplacental Doppler ratio <10th centile	11.5 (28/243)	2.1 (0.8–6.0)	0.15	2.7 (1.0–7.7)	0.053
Cerebroplacental Doppler ratio <1.0	11.5 (28/243)	1.4 (0.6–3.4)	0.48	3.3 (1.2–9.2)	0.024
Low birth weight (n = 67)^c^					
Umbilical artery resistance index >90th centile	16.6 (60/361)	1.3 (0.6–2.7)	0.52	1.3 (0.6–2.8)	0.56
Middle cerebral artery pulsatility index <10th centile	17.8 (53/298)	1.9 (0.9–4.1)	0.08	2.0 (1.0–4.3)	0.07
Cerebroplacental Doppler ratio <10th centile	17.8 (45/252)	2.8 (1.3–6.3)	0.011	2.5 (1.1–5.7)	0.033
Cerebroplacental Doppler ratio <1.0	17.8 (45/252)	2.6 (1.3–4.9)	0.005	2.8 (1.3–6.0)	0.007
Placental malaria (n = 46)^c^					
Umbilical artery resistance index >90th centile	15.6 (44/281)	4.9 (2.3–10.7)	<0.001	5.1 (2.3–10.9)	<0.001
Middle cerebral artery pulsatility index <10th centile	14.5 (34/234)	1.8 (0.7–5.1)	0.24	1.8 (0.6–5.0)	0.23
Cerebroplacental Doppler ratio <10th centile	14.7 (29/197)	1.5 (0.5–5.0)	0.47	1.3 (0.4–4.6)	0.65
Cerebroplacental Doppler ratio <1.0	14.7 (29/197)	1.8 (0.8–4.3)	0.16	1.8 (0.7–4.8)	0.26

* Adjusted for fetal sex, gravidity and gestational age

^a^N (%) represents the number of scans

^b^N (%) represents the number of women

A reduced CPR was associated with SGA (2.8, 95% CI 1.0–7.7, P = 0.053) and LBW (2.5, 1.01–5.7; P = 0.033), and similar results were observed when low CPR was defined as CPR <1.0 (Table 5). An increased UARI was associated with placental infection (5.1, 2.3–10.9, P < 0.001). However, an UARI <10th centile had comparatively low sensitivity for the detection of (occult) placental malaria (sensitivity: 0.26; positive predictive value: 0.41) but may be a useful tool to exclude women with placental infection (specificity: 0.93; negative predictive value: 0.87).

There was no effect measure modification of the association between malaria infection and UARI, MCAPI or CPR by characteristics such as MUAC <22 cm, BMI <18.5 kg/m^2^ or height <150 cm, risk behaviours such as smoking, drinking alcohol or betel nut chewing habits, or gravidity or fetal sex.

## Discussion

In this first Doppler ultrasound study from PNG to determine factors related to abnormal fetoplacental and intrafetal blood flow, peripheral *P. falciparum* parasitaemia was associated with an increased UARI and a decreased MCAPI. Associations were strongest when the impact of overall malaria exposure (peripheral infection during any stage of pregnancy) on flow was evaluated. Here, increased UARI and intrafetal flow redistribution were observed amongst women with microscopic or sub-microscopic *P. falciparum* infections. No such effects were observed for *P. vivax* infections or infections detected in early pregnancy (<20 gestational weeks) but their numbers were low. Documented episodes of increased UARI were associated with histological evidence of placental malaria at delivery.

The present study contributes further evidence that *P. falciparum* infection can have deleterious effects on fetoplacental flow and intrafetal blood flow redistribution. Concurrent *P. falciparum* infection in early pregnancy was associated with increased UARI in two studies from Kenya [16, 17], and women with symptomatic falciparum malaria had increased UARI in research from French Guiana [14]. An increased UARI may be indicative of some level of placental insufficiency, either temporary or permanent. Previously proposed mechanisms include negative effects of *P. falciparum* infection on placental vascular neogenesis, or potentially on uterine spiral artery invasion and conversion by reducing trophoblast motility and invasion capacity [5, 7]. Alternatively, *P. falciparum* infection may lead to more acute processes affecting placental function and perfusion, e.g., inflammation and placental thromboxane production. For the first time, the present research shows that falciparum malaria is associated with intrafetal blood flow redistribution, the diversion of blood flow from peripheral organs to more ‘essential’ organs, such as the brain. This can be a sonographic manifestation of growth restriction, and in clinical practice MCAPI is used to assess severity of suspected FGR in fetuses measuring SGA and who have normal umbilical artery Doppler studies [10]. Although malaria infection was not associated with SGA, malarial infection alters Doppler flow indices in a manner suggesting at least temporary placental insufficiency: anti-malarial treatment provided as part of the parent trial and unscheduled visits for ill health may have limited the establishment of the potentially more deleterious chronic placental infections.

Interestingly, malaria infection was not associated with measuring SGA in pregnancy or at birth in this cohort [25]. Most malaria infections were detected at enrolment, when all women received presumptive anti-malarial treatment. Clearance of infections at study enrolment may have limited their chronic effects on placental development and function [23]. Consistent with this, there were no differences in UARI or MCAPI by treatment arm (single treatment with SP and chloroquine or three doses of SP and AZ (see Additional files 2, 4, 5) or SGA [25]. Nevertheless, in the trial analysis active placental infection was reduced amongst women receiving SPAZ, and placental infection was a risk for LBW [23].

The present study observed stronger associations between malaria ‘exposure’ (peripheral infections detected at any stage during pregnancy) and Doppler indices than for malaria infections detected in peripheral blood at or before the Doppler study time point. The rationale for assessing associations of infections detected after ultrasound scan is that there is increasing evidence that in areas with declining malaria transmission a large number of infections remain undetected even by qPCR, in particular in pregnancy [46–48]. In addition, infection risk is clustered, i.e., the same individuals tend to get re-infected, and this is particularly common with *P. falciparum* [48]: women with malaria detection after scan are more likely to have been infected prior or at scan, than those who were not.

*Plasmodium vivax* infections have been previously associated with LBW, and research indicates that such infections also affect umbilical artery flow [18, 49]. The present study was unable to demonstrate an effect of vivax malaria on UA or MCA flow, possibly because there were few detectable *P. vivax* infections and the study lacked power to demonstrate such relationships. There was no observable effect measure modification of the *P. falciparum* parasitaemia-blood flow relationship by maternal nutritional status or gravidity, in contrast to what has been demonstrated in other similar studies [17, 50].

*Plasmodium falciparum* infection was associated with increased UA resistance in all analyses (infection before or at scan, any peripheral infection), yet the ‘exposure’ analysis revealed important additional associations, such as the impact of sub-microscopic *P. falciparum* infections on flow, with particularly strong impacts on the MCAPI and the CPR. Of note, a low CPR was the Doppler ultrasound measurement most strongly associated with LBW in this cohort. There is evidence to suggest that sub-microscopic *P. falciparum* infections can cause LBW, anaemia and possibly preterm birth [45, 46]. This is of great concern, as these infections are common and are frequently undetectable by rapid diagnostic tests. As such, strategies such as intermittent screening and treatment for malaria are likely to miss infections that are directly involved in causing fetal pathology and adverse pregnancy outcome.

In contrast to other cohorts, gravidity, fetal sex [18] and MUAC, height and anaemia [51] were not associated with UARI or MCAPI. Risk behaviour such as smoking tobacco, chewing betelnut and drinking alcohol did not statistically significantly increase the proportion of abnormal Doppler episodes, although smoking tobacco, as a risk factor for high UARI, approached significance (P = 0.085, Additional file 2) [52].

Women with increased UARI measurement during pregnancy were more likely to have evidence of placental infection on histology. Although UARI had low sensitivity (0.26) for the detection of placental malaria, it had a good negative predictive value (0.87). Where ultrasound is available, further evaluation of its ability to predict placental malaria infection is indicated. The sensitivity of UARI could be improved by combining this measurement with other simple investigations that may help identify women with occult placental malaria [53].

This study has some limitations. Firstly, the study was insufficiently powered to evaluate the effect of early pregnancy malaria and vivax malaria on fetoplacental and intrafetal flow. Secondly, the number of Doppler velocimetry measurements and malaria screening episodes was comparatively small, and thus episodes of abnormal flow or infection may have been missed. Only ~60 women had Doppler indices measured at two or more timepoints during pregnancy. In addition, only one measurement per assessment (UA, MCA) was taken during each scanning episode, instead of the recommended three measurements [10]. Furthermore, there was no external quality control for the MCA measurements, although all image stills for UA measurements randomly selected for review passed quality control. Thirdly, the study may not have measured and adjusted for all confounding factors, such as indoor pollution. Lastly, previously published reference ranges for MCAPI and CPR could not be used for analysis as distribution of these variables in the study population differed significantly from published reference standards [10, 11]. As such, a within-population analysis was performed deriving 10th/90th centiles using an established approach [44] and defining likely ‘abnormal’ Doppler indices as those outside of the 10th/90th centile boundaries. In addition, analyses were repeated using a categorical cut off of <1.0 to define an abnormal CPR. It is possible that the observed difference in population averages is due to the known lower blood pressures in rural PNG populations than those of Westernized populations [54, 55].

## Conclusion

In PNG, both microscopic and sub-microscopic *P. falciparum* infections in pregnancy are associated with increased UARI and reduced MCAPI, which are indicators of placental insufficiency and fetal blood flow redistribution, respectively, and are both associated with FGR. Malaria prevention strategies for pregnant women must be able to clear all infections, including sub-microscopic ones. In malaria-endemic areas an abnormal UARI could indicate presence of placental infection, and women may benefit from presumptive treatment with a highly effective anti-malarial.

## Additional files

**Additional file 1.** Umbilical artery resistance index (UARI, Panel A), middle cerebral artery pulsatility index (MCAPI, Panel B) and cerebroplacental Doppler ratio (CPR, Panel C) data from this study and reference ranges for change with gestational age from previous studies [10, 11]. While the UARI reference range overlaps significantly with the data collected in the present study, the MCAPI and CPR reference ranges are markedly different from the present data. This necessitated the derivation of population-specific definitions of abnormal Doppler velocimetry measurements, as described in “Methods” section.

**Additional file 2.** Malaria infection, nutritional factors, smoking, and other potential associations with elevated umbilical artery resistance index (UARI, >90th centile) during pregnancy in PNG women In contrast to other cohorts, nutritional factors such as MUAC, height and anaemia were not associated with increased UARI. Smoking tobacco, a known risk factor for elevated UARI approached significance (P = 0.085) while other risk behaviours, such as chewing betelnut and drinking alcohol, showed no association.

**Additional file 3.** Malaria infection, nutritional factors, smoking, and other potential associations with decreased middle cerebral artery pulsatility index (MCAPI, <10th centile) during pregnancy in PNG women. No significant differences were observed when various known nutritional and behavioural risk factors were assessed.

**Additional file 4.** Malaria infection, nutritional factors, smoking, and other potential associations with decreased cerebroplacental Doppler ratio (CPR, <10th centile) during pregnancy in PNG women. Cerebroplacental ratio was not significantly influenced by various nutritional, behavioural and other factors.

**Additional file 5.** Associations of recent malaria infection with Doppler indices. A total of 533 malaria tests preceded the Doppler scan by no more than 6 weeks and were thus considered ‘recent’. In this category, 17 were positive for *P. falciparum* with either method (13 by qPCR; 11 by LM) and two were positive for *P. vivax*, by qPCR. Of these, seven infections were sub-microscopic. These data are not shown in the main text as the numbers of recent malaria episodes were too low to confidently perform statistical analyses.

**Additional file 6.** Association of peripheral malaria infection with reduced cerebroplacental Doppler ratio (<1.0).

## Abbreviations

UA: umbilical artery
MCA: middle cerebral artery
CPR: cerebroplacental ratio
GEE: generalized estimating equations
FGR: fetal growth restriction
LBW: low birth weight
LMICs: low-and middle-income countries
UARI: umbilical artery resistance index
MCAPI: middle cerebral artery pulsatility index
SGA: small-for-gestational age
PNG: Papua New Guinea
IPTp: intermittent preventive treatment in pregnancy
SP: sulfadoxine pyrimethamine
AZ: azithromycin
Hb: haemoglobin
MUAC: mid-upper arm circumference
BW: birth weight
qPCR: quantitative polymerase chain reaction
hs-CRP: high sensitive C-reactive protein
HC: head circumference
FL: femur length
GA: gestational age
BMI: body mass index
LM: light microscopy
aOR: adjusted odds ratio
EFW: estimated fetal weight
AC: abdominal circumference
LM: light microscopy

## References

[1] Christian P, Lee SE, Donahue Angel M, Adair LS, Arifeen SE, Ashorn P (2013). Risk of childhood undernutrition related to small-for-gestational age and preterm birth in low- and middle-income countries. Int J Epidemiol.

[2] Wang H, Liddell CA, Coates MM, Mooney MD, Levitz CE, Schumacher AE (2014). Global, regional, and national levels of neonatal, infant, and under-5 mortality during 1990–2013: a systematic analysis for the global burden of disease study 2013. Lancet.

[3] Umbers AJ, Aitken EH, Rogerson SJ (2011). Malaria in pregnancy: small babies, big problem. Trends Parasitol.

[4] Huynh BT, Cottrell G, Cot M, Briand V (2014). Burden of malaria in early pregnancy: a neglected problem?. Clin Infect Dis.

[5] Umbers AJ, Stanisic DI, Ome M, Wangnapi R, Hanieh S, Unger HW (2013). Does malaria affect placental development? Evidence from in vitro models. PLoS ONE.

[6] Boeuf P, Aitken EH, Chandrasiri U, Chua CL, McInerney B, McQuade L (2013). *Plasmodium falciparum* malaria elicits inflammatory responses that dysregulate placental amino acid transport. PLoS Pathog.

[7] Conroy AL, Silver KL, Zhong K, Rennie M, Ward P, Sarma JV (2013). Complement activation and the resulting placental vascular insufficiency drives fetal growth restriction associated with placental malaria. Cell Host Microbe.

[8] Umbers AJ, Boeuf P, Clapham C, Stanisic DI, Baiwog F, Mueller I (2011). Placental malaria-associated inflammation disturbs the insulin-like growth factor axis of fetal growth regulation. J Infect Dis.

[9] Chandrasiri UP, Chua CL, Umbers AJ, Chaluluka E, Glazier JD, Rogerson SJ (2014). Insight into the pathogenesis of fetal growth restriction in placental malaria: decreased placental glucose transporter isoform 1 expression. J Infect Dis.

[10] Acharya G, Wilsgaard T, Berntsen GK, Maltau JM, Kiserud T (2005). Reference ranges for serial measurements of umbilical artery Doppler indices in the second half of pregnancy. Am J Obstet Gynecol.

[11] Ebbing C, Rasmussen S, Kiserud T (2007). Middle cerebral artery blood flow velocities and pulsatility index and the cerebroplacental pulsatility ratio: longitudinal reference ranges and terms for serial measurements. Ultrasound Obstet Gynecol.

[12] Mari G, Deter RL (1992). Middle cerebral artery flow velocity waveforms in normal and small-for-gestational-age fetuses. Am J Obstet Gynecol.

[13] McCowan LM, Harding JE, Stewart AW (2000). Umbilical artery Doppler studies in small for gestational age babies reflect disease severity. BJOG.

[14] Arbeille P, Carles G, Bousquet F, Body G, Lansac J (1998). Fetal cerebral and umbilical artery blood flow changes during pregnancy complicated by malaria. J Ultrasound Med.

[15] Arbeille P, Carles G, Georgescu M, Tobal N, Herault S, Bousquet F (2003). Consequences of reduced umbilical and increased foetal cerebral flow during malaria crisis on foetal behaviour. Parasitology.

[16] Dorman EK, Shulman CE, Kingdom J, Bulmer JN, Mwendwa J, Peshu N (2002). Impaired uteroplacental blood flow in pregnancies complicated by falciparum malaria. Ultrasound Obstet Gynecol.

[17] McClure EM, Meshnick SR, Lazebnik N, Mungai P, King CL, Hudgens M (2014). A cohort study of *Plasmodium falciparum* malaria in pregnancy and associations with uteroplacental blood flow and fetal anthropometrics in Kenya. Int J Gynaecol Obstet.

[18] Griffin JB, Lokomba V, Landis SH, Thorp JM, Herring AH, Tshefu AK (2012). *Plasmodium falciparum* parasitaemia in the first half of pregnancy, uterine and umbilical artery blood flow, and foetal growth: a longitudinal Doppler ultrasound study. Malar J.

[19] Machado Filho AC, da Costa EP, da Costa EP, Reis IS, Fernandes EA, Paim BV (2014). Effects of vivax malaria acquired before 20 weeks of pregnancy on subsequent changes in fetal growth. Am J Trop Med Hyg.

[20] Muller I, Bockarie M, Alpers M, Smith T (2003). The epidemiology of malaria in Papua New Guinea. Trends Parasitol.

[21] Stanisic D, Moore K, Baiwog F, Ura A, Clapham C, King C (2015). Risk factors for malaria and adverse birth outcomes in a prospective cohort of pregnant Papua New Guinea women. Trans R Soc Trop Med Hyg.

[22] Senn M, Baiwog F, Winmai J, Mueller I, Rogerson S, Senn N (2009). Betel nut chewing during pregnancy, Madang province, Papua New Guinea. Drug Alcohol Depend.

[23] Unger HW, Ome-Kaius M, Wangnapi RA, Umbers AJ, Hanieh S, Suen CS (2015). Sulphadoxine–pyrimethamine plus azithromycin for the prevention of low birthweight in Papua New Guinea: a randomised controlled trial. BMC Med.

[24] Allen SJ, Raiko A, O’Donnell A, Alexander ND, Clegg JB (1998). Causes of preterm delivery and intrauterine growth retardation in a malaria endemic region of Papua New Guinea. Arch Dis Child Fetal Neonatal Ed.

[25] Unger HW, Ome-Kaius M, Karl S, Singirok D, Siba P, Walker J (2015). Factors associated with ultrasound-aided detection of suboptimal fetal growth in a malaria-endemic area in Papua New Guinea. BMC Pregnancy Childbirth.

[26] Unger HW, Karl S, Wangnapi RA, Siba P, Mola G, Walker J (2014). Fetal size in a rural Melanesian population with minimal risk factors for growth restriction: an observational ultrasound study from Papua New Guinea. Am J Trop Med Hyg.

[27] Mueller I, Rogerson S, Mola GD, Reeder JC (2008). A review of the current state of malaria among pregnant women in Papua New Guinea. PNG Med J.

[28] Child mortality estimates Papua New Guinea. http://www.childmortality.org/. Accessed 15 May 2015.

[29] Loughna P, Chitty L, Evans T, Chudleigh T (2009). Fetal size and dating: charts recommended for clinical obstetric practice. Ultrasound.

[30] DOH. National malaria treatment protocol. Port Moresby: Papua New Guinea National Department of Health; 2009.

[31] Rijken MJ, Rijken JA, Papageorghiou AT, Kennedy SH, Visser GH, Nosten F (2011). Malaria in pregnancy: the difficulties in measuring birthweight. BJOG.

[32] Laman M, Moore BR, Benjamin JM, Yadi G, Bona C, Warrel J (2014). Artemisinin–naphthoquine versus artemether–lumefantrine for uncomplicated malaria in Papua New Guinean children: an open-label randomized trial. PLoS Med.

[33] Rosanas-Urgell A, Mueller D, Betuela I, Barnadas C, Iga J, Zimmerman PA (2010). Comparison of diagnostic methods for the detection and quantification of the four sympatric *Plasmodium* species in field samples from Papua New Guinea. Malar J.

[34] Rogerson SJ, Hviid L, Duffy PE, Leke RF, Taylor DW (2007). Malaria in pregnancy: pathogenesis and immunity. Lancet Infect Dis.

[35] Mongelli M, Wilcox M, Gardosi J (1996). Estimating the date of confinement: ultrasonographic biometry versus certain menstrual dates. Am J Obstet Gynecol.

[36] Karl S, Li Wai Suen CS, Unger HW, Ome-Kaius M, Mola G, White L (2015). Preterm or not—an evaluation of estimates of gestational age in a cohort of women from rural papua new Guinea. PLoS ONE.

[37] Hadlock FP, Harrist RB, Martinez-Poyer J (1991). In utero analysis of fetal growth: a sonographic weight standard. Radiology.

[38] Ververs MT, Antierens A, Sackl A, Staderini N, Captier V (2013). Which anthropometric indicators identify a pregnant woman as acutely malnourished and predict adverse birth outcomes in the humanitarian context?. PLoS Curr Disasters.

[39] Smith T, Vounatsou P (2003). Estimation of infection and recovery rates for highly polymorphic parasites when detectability is imperfect, using hidden Markov models. Stat Med.

[40] Barry AE, Schultz L, Senn N, Nale J, Kiniboro B, Siba PM (2013). High levels of genetic diversity of *Plasmodium falciparum* populations in Papua New Guinea despite variable infection prevalence. Am J Trop Med Hyg.

[41] Gosling RG, Dunbar G, King DH, Newman DL, Side CD, Woodcock JP (1971). The quantitative analysis of occlusive peripheral arterial disease by a non-intrusive ultrasonic technique. Angiology.

[42] Jacquemyn Y, Verdonk P (2001). Doppler ultrasound of the fetomaternal circulation: a preliminary study on differences between ethnic groups. Clin Exp Obstet Gynecol.

[43] Prior T, Wild M, Mullins E, Bennett P, Kumar S (2013). Sex specific differences in fetal middle cerebral artery and umbilical venous Doppler. PLoS ONE.

[44] Royston P (1995). Calculation of unconditional and conditional reference intervals for foetal size and growth from longitudinal measurements. Stat Med.

[45] Cottrell G, Moussiliou A, Luty AJ, Cot M, Fievet N, Massougbodji A (2015). Submicroscopic *Plasmodium falciparum* infections are associated with maternal anemia, premature births, and low birth weight. Clin Infect Dis.

[46] Cohee LM, Kalilani-Phiri L, Boudova S, Joshi S, Mukadam R, Seydel KB (2014). Submicroscopic malaria infection during pregnancy and the impact of intermittent preventive treatment. Malar J.

[47] Bousema T, Okell L, Felger I, Drakeley C (2014). Asymptomatic malaria infections: detectability, transmissibility and public health relevance. Nat Rev Microbiol.

[48] Bousema T, Griffin JT, Sauerwein RW, Smith DL, Churcher TS, Takken W (2012). Hitting hotspots: spatial targeting of malaria for control and elimination. PLoS Med.

[49] Nosten F, McGready R, Simpson JA, Thwai KL, Balkan S, Cho T (1999). Effects of *Plasmodium vivax* malaria in pregnancy. Lancet.

[50] Landis SH, Lokomba V, Ananth CV, Atibu J, Ryder RW, Hartmann KE (2009). Impact of maternal malaria and under-nutrition on intrauterine growth restriction: a prospective ultrasound study in Democratic Republic of Congo. Epidemiol Infect.

[51] Belkacemi L, Nelson DM, Desai M, Ross MG (2010). Maternal undernutrition influences placental-fetal development. Biol Reprod.

[52] Pfarrer C, Macara L, Leiser R, Kingdom J (1999). Adaptive angiogenesis in placentas of heavy smokers. Lancet.

[53] Chua CL, Robinson LJ, Baiwog F, Stanisic DI, Hamilton JA, Brown GV (2014). High numbers of circulating pigmented polymorphonuclear neutrophils as a prognostic marker for decreased birth weight during malaria in pregnancy. Int J Parasitol.

[54] Benjamin AL (2006). Community screening for high blood pressure among adults in urban and rural Papua New Guinea. P N G Med J.

[55] Maddocks I, Rovin L (2005). A New Guinea population in which blood pressure appears to fall as age advances. P N G Med J.

