# Supplementary figures and images for: Effects of Plasmodium falciparum infection on umbilical artery resistance and intrafetal blood flow distribution: a Doppler ultrasound study from Papua New Guinea
Source: Malar J. 2017 Jan 19;16:35. doi: 10.1186/s12936-017-1689-z (PMC5248505; doi:10.1186/s12936-017-1689-z)

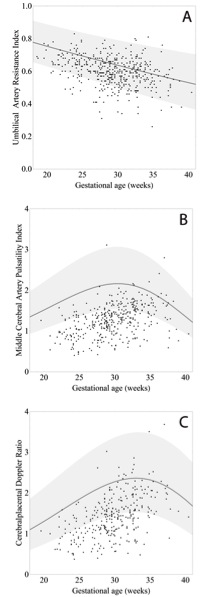

Supplement: Supplementary file 1 — Additional file 1. Umbilical artery resistance index (UARI, Panel A), middle cerebral artery pulsatility index (MCAPI, Panel B) and cerebroplacental Doppler ratio (CPR, Panel C) data from this study and reference ranges for change with gestational age from previous studies [10, 11]. While the UARI reference range overlaps significantly with the data collected in the present study, the MCAPI and CPR reference ranges are markedly different from the present data. This necessitated the derivation of population-specific definitions of abnormal Doppler velocimetry measurements, as described in “Methods” section. [file 12936_2017_1689_MOESM1_ESM.jpg]
