# Supplementary material for: Effects of Plasmodium falciparum infection on umbilical artery resistance and intrafetal blood flow distribution: a Doppler ultrasound study from Papua New Guinea
Source: Malar J. 2017 Jan 19;16:35. doi: 10.1186/s12936-017-1689-z (PMC5248505; doi:10.1186/s12936-017-1689-z)
Supplement: Supplementary file 3 — Additional file 3. Malaria infection, nutritional factors, smoking, and other potential associations with decreased middle cerebral artery pulsatility index (MCAPI, <10th centile) during pregnancy in PNG women. No significant differences were observed when various known nutritional and behavioural risk factors were assessed. [file 12936_2017_1689_MOESM3_ESM.doc]

**Additional file 6** Association of peripheral malaria infection with reduced cerebroplacental Doppler ratio (<1.0)

|  | % (N) | OR (95%CI) | P | aOR (95%CI)* | P |
| --- | --- | --- | --- | --- | --- |
| *Any malaria infection prior to Doppler scan*** | | | | | |
| **Any species** | 14.8 (45/303) | 0.8 (0.3-1.9) | 0.59 | 0.9 (0.3-2.3) | 0.8 |
| ***P.f.*** | 12.5 (38/303) | 1.0 (0.4-2.) | 1.00 | 1.0 (0.4-2.9) | 1.00 |
| ***P.v*.** | 3.3 (10/303) | - | - | - | - |
| ***P.f*. (sub)** | 5.7 (17/296) | 1.6 (0.5-4.7) | 0.8 | 1.6 (0.4-6.4) | 0.49 |
|  | | | | | |
| *Peripheral malaria infection at any stage in pregnancy (as a measure of exposure)**** | | | | | |
| **Any species** | 21.4 (54/252) | 1.0 (0.5-2.1) | 0.96 | 1.1 (0.5-2.5) | 0.78 |
| ***P.f*.** | 17.1 (43/252) | 1.4 (0.7-3.1) | 0.34 | 1.5 (0.6-3.4) | 0.39 |
| ***P.v.*** | 7.1 (18/252) | 0.6 (0.1-3.0) | 0.57 | 0.8 (0.17-3.7) | 0.77 |
| ***P.f*. (sub)** | 8.2 (20/244) | 2.4 (0.9-6.6) | 0.09 | 2.5 (0.7-8.5) | 0.14 |
|  | | | | | |
| *Cumulative malaria detections with any species (as a measure of exposure)*** | | | | | |
| **1** | 17.9 (45/252) | 0.6 (0.3-1.4) | 0.25 | 0.6 (0.2-1.6) | 0.32 |
| **≥2** | 3.6 (9/252) | 4.5 (1.1-18.7) | 0.04 | 5.5 (1.2-24.0) | 0.024 |

**Note:** *P.f*., *P. falciparum*; *P.v*., *P. vivax*; sub: sub-microscopic;

*adjusted for fetal sex, gravidity and gestational age;

** N(%) represents the number of scans;

*** N(%) represents the number of women.
